# Autoimmune Neuroinflammatory Diseases: Role of Interleukins

**DOI:** 10.3390/ijms24097960

**Published:** 2023-04-27

**Authors:** Abdul Waheed Khan, Mariya Farooq, Moon-Jung Hwang, Muhammad Haseeb, Sangdun Choi

**Affiliations:** 1 Department of Molecular Science and Technology, Ajou University, Suwon 16499, Republic of Korea; 2 S&K Therapeutics, Ajou University Campus Plaza 418, 199 Worldcup-ro, Yeongtong-gu, Suwon 16502, Republic of Korea

**Keywords:** interleukins, multiple sclerosis, amyotrophic lateral sclerosis, Alzheimer disease, neuromyelitis optica, autoimmune encephalitis, epidemiology

## Abstract

Autoimmune neuroinflammatory diseases are a group of disorders resulting from abnormal immune responses in the nervous system, causing inflammation and tissue damage. The interleukin (IL) family of cytokines, especially IL-1, IL-6, and IL-17, plays a critical role in the pathogenesis of these diseases. IL-1 is involved in the activation of immune cells, production of pro-inflammatory cytokines, and promotion of blood-brain barrier breakdown. IL-6 is essential for the differentiation of T cells into Th17 cells and has been implicated in the initiation and progression of neuroinflammation. IL-17 is a potent pro-inflammatory cytokine produced by Th17 cells that plays a crucial role in recruiting immune cells to sites of inflammation. This review summarizes the current understanding of the roles of different interleukins in autoimmune neuroinflammatory diseases, including multiple sclerosis, amyotrophic lateral sclerosis, Alzheimer’s disease, neuromyelitis optica, and autoimmune encephalitis, and discusses the potential of targeting ILs as a therapeutic strategy against these diseases. We also highlight the need for further research to better understand the roles of ILs in autoimmune neuroinflammatory diseases and to identify new targets for treating these debilitating diseases.

## 1. Introduction

Neuroinflammation is a complex process involving the activation of immune cells and release of pro-inflammatory mediators within the central nervous system (CNS) in response to injury, infection, or neurodegeneration. Acute inflammation is essential for resolving injuries or infections; however, chronic neuroinflammation can cause tissue damage and neuronal loss, contributing to the pathogenesis of various neurological disorders [1].

In autoimmune neuroinflammatory diseases, the immune system attacks healthy nerve cells and tissues, resulting in inflammation and damage. Such disorders can affect the brain, spinal cord, and peripheral nerves and produce a variety of symptoms [2]. The exact causes of autoimmune neuroinflammatory diseases are yet to be ascertained; however, a combination of genetic and environmental factors is believed to contribute to their development. In some cases, infections or exposure to certain medications or toxins may trigger an autoimmune response [3].

Interleukins (ILs) are signaling proteins that facilitate interactions with both immune and non-immune cells [4]. They were formerly believed to be exclusively expressed by leukocytes; however, a variety of other cells, including monocytes, endothelial cells, and macrophages, have also been found to produce ILs [5]. They are essential for the activation, differentiation, proliferation, maturation, migration, and adhesion of immune cells. The primary function of ILs is to control cell proliferation, differentiation, and activation during immunological and inflammatory responses. They perform both paracrine and autocrine functions [6]. Interleukins may be pro-inflammatory, as shown in Table 1.

ILs help to regulate immune and inflammatory responses. ILs play crucial roles in the pathogenesis of various autoimmune and inflammatory disorders, including psoriasis, inflammatory bowel disease, and rheumatoid arthritis [6]. Recently, interleukins have also been found to play a crucial role in the pathogenesis of neurological diseases associated with neuroinflammation.

Research on autoimmune neuroinflammatory diseases is ongoing, and novel therapeutic measures are under development. The ultimate goal is to improve the quality of life of patients with these conditions and develop a cure. The clinical drugs targeting interleukins for treating neuroinflammatory diseases [17] are shown in Table 2.

**Figure 1:**
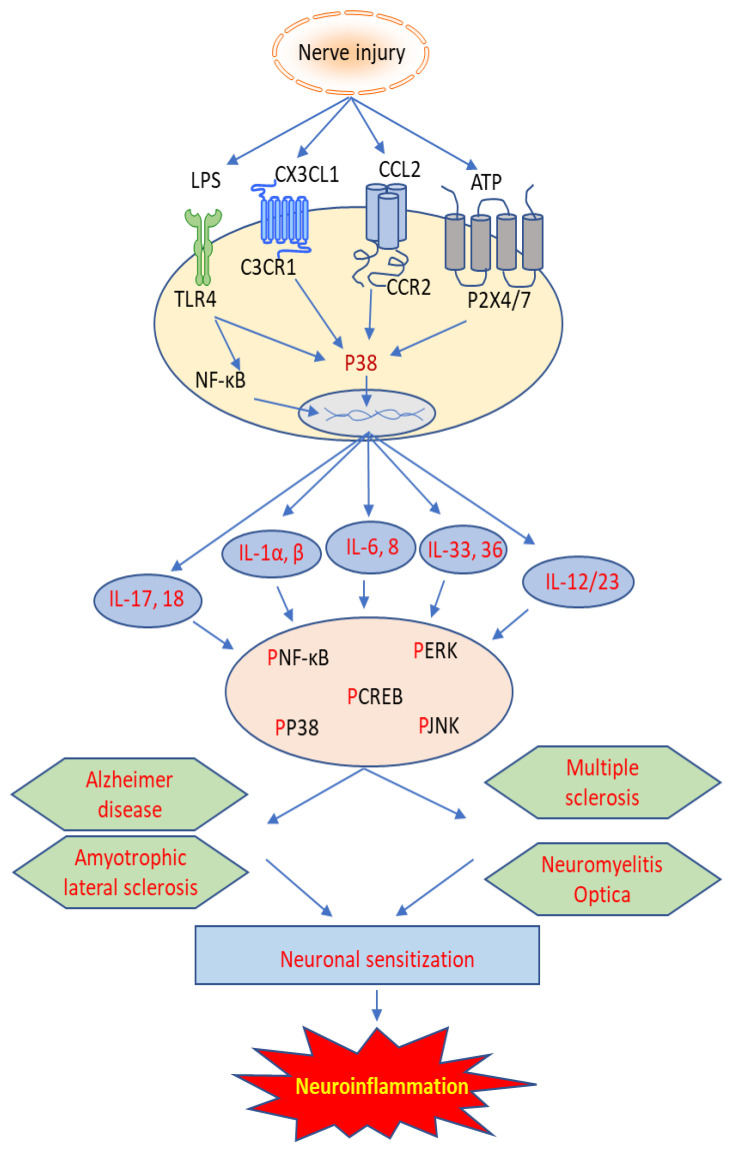
Cellular mechanism of interleukins involved in autoimmune neuroinflammatory diseases.

This paper aims to review the current knowledge regarding the role of IL family members in various neurological diseases, including multiple sclerosis (MS), Alzheimer’s disease (AD), amyotrophic lateral sclerosis (ALS), neuromyelitis optica (NMO), and autoimmune encephalitis (AE). As is shown in Figure 1, the involvement and mechanism of interleukins in neuroinflammation may yield novel targets for treating these devastating diseases.

### 1.1. Prevalence and Epidemiology of Neuroinflammatory Diseases

The precise epidemiology of neuroinflammatory diseases is not well-defined, and estimates vary depending on specific conditions. MS is one of the most well-known neuroinflammatory diseases and affects approximately 2.8 million people globally [18]. The global prevalence of MS is approximately 33.8 cases per 100,000 people, with significant regional variations. For example, its prevalence is higher in Europe (95.2/100,000 cases) and North America (70.9 cases per 100,000) than in Asia (3.3 cases per 100,000) and Africa (2.3 cases per 100,000) [19]. MS is more commonly diagnosed in women than in men, with a female-to-male ratio of approximately 2:1 [20]. The age of onset is usually between 20 and 40 years, although it can occur at any age [21]. The epidemiology of ALS is complex, and the precise cause of the disease remains unclear. ALS occurs sporadically in most cases; however, a small proportion of cases are familial, with mutations in several genes linked to ALS. The prevalence of ALS is estimated to be five cases per 100,000 individuals worldwide, with incidence rates ranging from 1.5 to 2.7 cases per 100,000 individuals annually [22]. The incidence and prevalence of ALS increases with age, and the disease is more common in men than in women.

According to the World Health Organization, approximately 50 million people worldwide are affected by dementia, with AD being the most common, accounting for 60–70% of cases [23]. The prevalence of AD increases with age, with most cases occurring in individuals over 65 years. In the United States, approximately 6.2 million individuals are living with AD, and this number is projected to increase to 13.8 million by 2050 [24]. NMO is less common but is increasingly being recognized and diagnosed. NO has a prevalence of approximately 0.5–4.4 per 100,000 individuals [25]. AE is a rare brain autoimmune disorder that is estimated to be found in 1/100,000 population [26]. 

### 1.2. Involvement of Interleukins in Neuroinflammatory Diseases

#### Multiple Sclerosis

Multiple sclerosis is a chronic neuroinflammatory disease characterized by nervous system lesions that can cause substantial neurological, cognitive, or physical impairment. Although the exact pathophysiology of MS remains unknown, existing data show that the pathogenesis of MS is multifactorial and includes both genetic and environmental factors, such as vitamin deficiencies, contact with infectious pathogens, and smoking. These substances can set off a series of immune system reactions that result in neurotoxicity, neuronal dysfunction, and nerve demyelination [27,28]. 

Among the inflammatory components, ILs are a large group of cell-secreted cytokines that bind to specific receptors. Leukocytes facilitate the induction and maintenance of diseases, resulting in tissue damage that affects demyelination and neuronal degeneration. ILs are pivotal in triggering and modulating the immune responses in MS [29].

Interleukins like IL-6, IL-17, and Il-1β are pro-inflammatory cytokines that stimulate immune cell activation and further cytokine production. The demyelination and damage seen in MS can be triggered by cytokines that contribute to the disruption of the blood-brain barrier and the recruitment of immune cells to the central nervous system, resulting in a wide spectrum of symptoms [30,31].

**Interleukin-6:** IL-6 is released by two different pathways: T cell-dependent release of IL-6 and T cell-independent IL-6 production. In the latter mechanism, the glial cells could influence the release of IL-6, as both brain macrophages and astrocytes secrete substantial amounts of IL-6 when infected with the virus or TNFα.

IL-6 is an important interleukin involved in MS pathogenesis. In an immunohistochemical study, IL-6 was found in both acute and chronic active plaques obtained from the brains of patients with MS. IL-6 was evenly distributed throughout the lesions in acute plaques, whereas in chronic conditions, it accumulated in regions of ongoing inflammation, such as lesion borders. Excess levels of IL-6 in brain tissue indicate its involvement in MS [32]. Additionally, using the cerebrospinal fluid (CSF) and serum samples taken from MS patients of different stages, IL-6 was found to be the major biomarker in MS, with the highest levels observed in advanced stage MS, that is, secondary progressive MS, compared to primary progressive and relapsingremitting MS [33]. 

In a study of an Experimental Autoimmune Encephalomyelitis (EAE) mouse model, IL-6-deficient mice injected with MOG developed milder EAE at a significantly lower frequency than wild-type animals [34], while administration of MR16-1, an anti-IL-6R antibody, improved mechanical allodynia and spontaneous neuropathic pain in EAE animals via inhibiting microglial activation and the descending pain inhibitory system [35]. In a clinical trial, tocilizumab (an anti-IL-6 receptor antibody) showed potential efficacy for treating patients with RRMS and seronegative NMOSD [36]. This indicates that IL-6 plays a major role in MS.

**Interleukin-17:** Recently, IL-17-secreting T cells (Th17 cells) were found to contribute to CNS demyelination and inflammation. According to the results published by a previous study, blood levels of IL-17A and IL-17F were significantly higher in patients with MS than in healthy controls, and IL-17F serum levels and the frequency of relapses were strongly correlated. The study concluded that elevated serum levels of IL-17A, particularly IL-17F, may enhance the risk of MS. Both IL-17 members stimulate the synthesis of local chemokines to attract monocytes and neutrophils to inflammatory sites, which worsens the disease [37].

A study was conducted to investigate the higher level of IL-17 in MS in IL-17^−/−^ murine disease models. A rodent model of multiple sclerosis (EAE) is significantly suppressed in IL-17 deficient mice. The Il-17 deficient mice showed delayed disease onset, improved histological changes, increased severity scores, and early recovery. This confirmed the involvement of IL-17 in MS pathology [38].

In vitro research, preclinical animal models, and intriguing results using the anti-IL-17A antibody (secukinumab) in small proof-of-concept studies in humans have shown signs of recovery in the rate of new gadolinium-enhanced lesions in patients with relapsing-remitting MS. This suggests that IL-17A, a major interleukin associated with autoimmune and inflammatory diseases, might be involved in MS [39]. 

**Interleukin-22:** IL-22 modifies immunity at the barrier surface in many human diseases. IL-22, together with IL-17, weakens the integrity of the blood-brain barrier and allows lymphocytes to enter the CNS, increasing the severity of MS [40].

Previously, IL-22 was present and colocalized with the IL-22 receptor on astrocytes, supporting the idea that IL-22 specifically targets astrocytes. However, whether the IL-22 found was produced by resident CNS cells, such as astrocytes, in an autocrine manner, or the IL-22 was of lymphocytic origin remains unclear. Here, IL-22 synthesis by CNS resident cells rather than release by T cells was slightly supported by the finding that IL-22 was not found in the CSF. However, because IL-22BP was discovered in the CSF, IL-22 in the CSF may also be trapped by this substance. This demonstrates the key role of IL-22 in the immunopathogenesis of MS [41].

In a human study, patients with relapsing-remitting multiple sclerosis (RRMS) were examined for IL-22 levels in their sera at various time intervals with or without treatment using interferon β-1a, interferon β-1b, and fingolimod. These findings demonstrated that the sera of untreated RRMS patients had significantly higher levels of IL-22 than those of healthy individuals. Fascinatingly, interferon β-1b, interferon β-1a, and fingolimod therapies considerably reduced serum concentrations of IL-22 at 6–12 months of treatment compared with their initial concentrations before starting treatment. The results of the correlation study showed a positive link between changes in the extended disability status score and changes in serum IL-22 concentration. These findings suggest that serum IL-22 levels may serve as a possible indicator of MS severity and treatment effectiveness [42].

**Interleukin-1β:** IL-1β is a strong pro-inflammatory cytokine that modulates the immune system in a variety of immunological linked diseases, including multiple sclerosis. IL-1β, which is produced by monocytes, microglial cells, astrocytes, and cerebral endothelial cells, plays a crucial role in the neuroinflammatory pathway of MS. By this effect, IL-1β facilitates activated leukocytes to cross the endothelium and enter the CNS. The role of IL-1β and its particular genotype in the development of MS has been extensively studied [43]. White matter pathology is primarily recognized for MS. However, new data suggest the presence of lesions in gray matter. IL-1β is one of the main mediators of inflammatory processes and has been linked to the etiology of MS.

The IL-1β involvement in MS was studied by Prins et al. They observed that the presence of IL-1β and IL-1ra-expressing cells in white matter regions of the brain in their experimental MS model was consistent with increased IL-1β [44] and IL-1ra [45] expression in active white matter lesions in post-mortem brain material of MS patients and of the animal EAE model for MS. Additionally, the production of IL-1β and IL-1ra within the ventricular choroid plexus is consistent with findings showing the early stages of MS in mice and causes a large increase in IL-1β production within the choroid plexus. Moreover, the CSF of MS patients had much higher amounts of the proteins IL-1β and IL-1ra. IL-1β and IL-1ra produced by the choroid plexus could be secreted into the CS, since it monitors the composition of the CSF content. This high concentration of IL-1β in the brain tissue proved the involvement of IL-1β in MS [46].

## 2. Amyotrophic Lateral Sclerosis

The neurodegenerative disease ALS, also known as Lou Gehrig’s disease, is a lethal neuronal disorder characterized by impaired motor neurons in the brain and spinal cord. Most patients die within 2–5 years of diagnosis because of respiratory distress caused by the loss of motor neurons [47,48]. Neuroinflammation is a prominent pathological hallmark in ALS. Additionally, infiltration of immune cells, including macrophages and T cells, at the sites of motor neuron damage has been observed in the CNS of patients with ALS. Clinical investigations have revealed that peripheral inflammation, including the presence of abnormal T cells, chemokines, cytokines, and other inflammatory biomarkers, is associated with ALS [49].

Researchers are increasingly focusing on the irregularities in peripheral blood chemokines and cytokines in ALS to further understand the origin of the disease, develop early detection methods, and perhaps even develop disease-modifying therapies. Interleukins play a major role in the pathogenesis of ALS via different mechanisms of action.

Microglia are immune cells found in the CNS, and several studies have linked interleukins to their activation. In ALS, microglia are involved in the neuroinflammatory response and have been linked to motor neuron loss. Disturbance in the level of several interleukins like pro-inflammatory IL-6 and anti-inflammatory Il-10 may result in the activation of microglia, resulting in neuroinflammations in ALS [50,51].

**Interleukin-6:** High concentrations of IL-6 have been observed in autoimmune and chronic inflammatory diseases.

Previously, plasma samples were collected from ALS patients and healthy controls to determine the role of IL-6 in ALS. Human bone marrow-derived endothelial cells (hBMECs) were used to study the impact of plasma proteins from patients with ALS and control subjects on endothelial cell (ECs) homeostasis and the immunoexpression of IL-6R and occludin (a tight junction protein) in endothelial cells. hBMECs showed signs of disorganization, including swelling, the formation of many cytoplasmic vesicles, and a decrease in cell processes two days after adding 10% ALS patient plasma to the culture medium. This was supported by the results of other experiments, where postmortem tissues from ALS showed vacuolated and enlarged ECs in the capillaries of the medulla, cervical, and lumbar spinal cord. To determine the exact IL-6 involvement in these changes, hBMECs were cultured and treated for 5 days with 10% plasma obtained from patients with ALS and controls. Immunohistochemical staining for occludin and IL-6R was performed using rabbit polyclonal anti-occludin antibodies and IL-6R mouse monoclonal antibodies, respectively, followed by incubation with a suitable secondary antibody. Using the ImageJ software (National Institutes of Health, Bethesda, MD, USA, https://imagej.nih.gov/ij/, version 1.46), the integrated density of positive cell expression per area in immunohistochemical images was measured to assess the fluorescence immunoexpression of IL6 and occludin. Plasma exposure in patients with ALS significantly increased IL-6R immunoexpression in ECs compared to the plasma of healthy individuals [52].

In an inhibitory study of IL-6, an ELISA of peripheral blood mononuclear cells obtained from patients with ALS and healthy controls was performed, and IL-6 expression levels in the serum of patients with ALS were compared with those of controls. High expression of IL-6 was detected in ALS patient samples compared to the control, which was suppressed using an IL-6 receptor antibody (tocilizumab), resulting in decreased inflammation [53]. 

**Interleukin-8:** The etiology of several neurodegenerative disorders (including AD, MS, and ALS) has been linked to inflammation involving cytokines and chemokines. The chemokine IL-8 is believed to be involved in the pathophysiology of neurodegenerative illnesses.

Previously, the concentrations of IL-8 in the CSF of patients with ALS were measured and compared with those in patients with other non-inflammatory neurological diseases (such as AD and Parkinson’s disease), with those of patients with MS as positive controls. Compared to patients with other non-inflammatory neurological disorders, patients with ALS had much greater amounts of IL-8 in their CSF, which were similar to the levels in patients with MS, indicating the involvement of IL-8 in immunological neurodegenerative diseases [54].

Another study was conducted using blood samples from patients with ALS and healthy controls. Two visits were conducted to collect blood samples from the participants and the concentrations of different inflammatory and antioxidant agents in the blood samples were determined. IL-8 concentration in the blood samples of patients were significantly higher than that in normal individuals, and its levels were higher at the second visit, while the antioxidant agent glutathione levels were found to be lower than that of normal individuals. This increase in IL-8 level shows the involvement of ALS, which increased with the passage of time (second visit). This shows that IL-8 plays a key role in disease pathology and its progression [55].

**Interleukin-10:** IL-10 is an anti-inflammatory interleukin whose expression increases during the primary stage of an inflammatory disease to counter inflammation. Its levels in the body also decrease with age, which is why inflammatory diseases, such as AD and ALS, are more common in the later stages of life [14]. Reactive microgliosis is a characteristic of severe ALS; however, its involvement in disease initiation remains uncertain.

Using a mouse model for live imaging of microglial activation crossed with SOD1G93A and SOD1G37R animal models of superoxide dismutase 1 (SOD1)-mutant-mediated ALS, the pre-onset phase of SOD1-mediated ALS was found to be characterized by a unique anti-inflammatory profile and a decreased innate immune/TLR2 response to LPS stimulation. This microglial phenotype was associated with a 16-fold increase in anti-inflammatory IL-10 levels following LPS exposure. Infusion of an IL-10R-blocking antibody at day 60 elevated microglial activation markers and accelerated ALS onset, but viral vector-mediated overexpression of IL-10 in microglial cells dramatically delayed disease onset and increased the survival of SOD1G93A mice. Elevated IL-10 levels in early ALS microglia were proposed to constitute a homeostatic and compensating “adaptive immune escape” mechanism that non-neuronally determined illness onsets [56].

**Interleukin-17:** Th17 cells are a newly discovered group of T cells that primarily produce IL-17 and are thought to play a key role in the pathogenesis of ALS and damage to autoimmunity. Previously, ELISA was used to measure the levels of IL-17 in serum and CSF samples taken from patients with ALS and other non-inflammatory neurological diseases (NIND) as a control group. IL-17 levels were significantly higher in both serum (*p* < 0.015) and CSF (*p* < 0.0006) samples from ALS patients as compared to those of patients with NIND. The overexpression of IL-17 in both the serum and CSF samples of patients showed that IL-17 may play a role in the pathogenic processes that lead to Th17 cell activation in ALS [57].

IL-17 expression was also evaluated in control subjects without autoimmune disorders and in patients with different types of ALS using ELISA. The serum concentrations of IL-17 were increased in patients with sporadic ALS (>5767 pg/mL) and familial ALS (>937 pg/mL) as compared to the control subjects (7 pg/mL). This showed that IL-17 is involved in ALS, and its level is directly related to the severity of ALS [58].

## 3. Alzheimer’s Disease

Pathological markers of AD include loss of neurons, neurofibrillary tangles, accumulation of activated glial cells, gliosis, and amyloid deposition, which result in senile plaques [59]. A pivotal event in the pathophysiology of AD is thought to be the imbalance between the development of amyloid (Aβ), a proteolytic component of amyloid precursor protein (APP), and its clearance [60,61]. The transition of Aβ from monomer to oligomer results in the formation of a key element of senile plaque, called insoluble fibrillar Aβ (fAβ). fAβ was thought to be the principal entity responsible for AD; however, new evidence reveals that soluble oligomeric amyloid (oAβ) discovered in the cortex of AD patients promotes AD development. The severity of cognitive impairment and degree of synapse loss are directly proportional to the amount of Aβ in the CSF or AD brain [62]. 

Although ILs have been implicated in the pathophysiology of AD, the exact processes by which they contribute to the development of the disease remain unclear. However, research suggests that AD development and progression can be aided by persistent brain inflammation caused by dysregulation of ILs. Pro-inflammatory cytokines, chemokines, and adhesion molecules are induced in the brain by inflammatory mediators such as interleukin-1β, IL-6, IL-18, etc. Neuronal damage and dysfunction, as well as the formation of Aβ plaques and tau tangles, might result from this persistent inflammation [63]. 

**Interleukin-1:** IL-1 is a multipotent immunoregulatory cytokine that plays a pivotal role in peripheral cellular and humoral immunity [64]. IL-1 is overexpressed in the brain of patients with AD, and this overexpression is strongly connected to plaque development and progression, senseless expansion of dystrophic neurites, and increased expression of acetylcholinesterase in neurons. Numerous effects of IL-1 have been linked to AD, including increased levels of neuronal Aβ precursor protein and other proteins associated with plaques, promotion of astrocyte activation, and astrocytic upregulation of S100B [65]. These latter occurrences could be connected to the excessive development of dystrophic neurites in neuritic plaques, an event required for transforming diffuse Aβ deposits into the neuritic amyloid plaques that are characteristic of AD. Recent genetic studies have confirmed the importance of IL-1 in AD pathogenesis via demonstrating that homozygosity for one particular polymorphism in the IL-1A gene triples the risk of developing AD, particularly for individuals with earlier disease onset and when combined with homozygosity for another polymorphism in the IL-1B gene [66].

A leading hypothesis for the mechanism driving neuroinflammation in AD is IL-1. In a mouse model of AD, researchers presented a transgenic mouse model with constant IL-1 overexpression, which triggered severe neuroinflammation that lasted for months following transgene activation. Astrocytic and microglial activation and the production of pro-inflammatory cytokines are characteristics of this reaction. Surprisingly, 4 weeks of IL-1 overexpression reduced amyloid pathology in the hippocampus of the APPswe/PS1dE9 animal model of AD. Insoluble amyloid beta 40 (Aβ40), Aβ42, frequency, and area portion of congophilic plaques were decreased dramatically. These findings suggest an adaptive function of IL-1-driven neuroinflammation in AD and may shed light on the recent failures of anti-inflammatory treatments for this condition [66].

In one study, investigators aimed to determine whether inhibiting IL-1 signaling has any effect on AD treatment in mice and, if so, via what molecular processes. According to their findings, prolonged administration of an IL-1R blocking antibody to 3xTg-AD mice considerably altered brain inflammatory responses, improved cognition, markedly slowed tau pathology, and partially decreased several fibrillar and oligomeric forms of amyloid. A reduction in NF-B activity correlates with changes in inflammatory responses. Additionally, suppression of IL-1 signaling decreases the levels of phosphorylated tau and the activity of many tau kinases in the brain, including GSK-3, cdk5/p25, and p38-MAPK. Additionally, we found that neuronal Wnt/β-catenin signaling and the cytokine S100B produced by astrocytes were decreased in 3xTg-AD brains. We also provide in vitro evidence that these changes may, in part, establish a mechanistic connection between IL-1 signaling and GSK-3 stimulation. Combining these findings, researchers have hypothesized that one of the major disease processes underlying AD may involve the IL-1 signaling cascade [67].

**Interleukin-6:** The development and differentiation of neurons in both the peripheral and central nervous systems are significantly influenced by the inflammatory cytokine IL-6. IL-6 promotes microglial activation, which leads to the creation of acute-phase proteins and phosphorylation of tau proteins in neurons. IL-6 recruits and stimulates microglia and astrocytes in the AD brain, releasing pro-inflammatory cytokines. This implies that IL-6 is crucial for brain inflammation and may be critical in the pathophysiology of AD [68].

In an investigational study, an AD mouse model was used to examine the IL-6 transition. The AD-induced mouse mortality was partially reversed by inhibiting IL-6 trans-signaling. It restored AD-induced alterations in exploration and anxiety before amyloid plaques were formed but did not affect locomotion in mice. However, following plaque formation, only locomotion was affected by suppressing IL-6 transcription. The amyloid plaque burden in the brain and hippocampus, and the levels of Aβ40 and Aβ42 in the cortex, of mice were likewise reduced when IL-6 trans-signaling was inhibited. Instead of neuroinflammation, the aforementioned changes may be due to changes in blood vessels and matrix organization. These findings strongly imply that IL-6 trans-signaling inhibition may be a potential therapeutic option for AD [69].

A study was conducted to assess the serum levels of IL-6 in patients with AD and examine the relationship between IL-6 and AD. ELISA was performed to determine the presence of IL-6 in the sera of 47 patients with clinically confirmed AD (27 women, 20 men) and 47 controls (25 women, 22 men). IL-6 levels in patients were 234 pg/mL, which was substantially higher than that in the control group (67 pg/mL); *p* < 0.001. This study clearly shows that patients with AD produce more IL-6, suggesting that these patients have impaired cellular immunity. The etiology of AD involves IL-6. These findings imply that acute-phase proteins found in the blood of patients with AD may be caused by elevated peripheral IL-6 secretion levels. These findings are encouraging for treating AD [70]. 

**Interleukin-18:** IL-18 is a pro-inflammatory cytokine involved in the pathogenesis of various neurodegenerative disorders, including AD. Increased levels of IL-18 were reported in the brain tissues and CSF of patients with AD compared to healthy controls, suggesting that IL-18 may play a role in inflammatory processes that contribute to AD pathology [71].

IL-18 has been shown to increase the production and deposition of Aβ in the brains of AD mice by promoting the expression of APP and β-site APP cleaving enzyme 1 (BACE1) that may contribute to the development of AD pathology by promoting Aβ accumulation. IL-18 binding protein was found to reduce Aβ accumulation and improve cognitive function in AD mice. The induction of BACE-1, APP processing, and Aβ by IL-18 is likely associated with stress-related neuronal adaptations during normal neuronal function and development. The effects of elevated or prolonged levels of IL-18 may contribute to AD progression, including via an increase in Aβ, leading to broader changes in the aging brain, especially in AD [72]. 

**Interleukin-33:** IL-33 is a cytokine that regulates immune responses and inflammatory processes.

Recently, IL-33 was found to play a role in AD pathogenesis by modulating neuroinflammation and synaptic plasticity. Previously, the expression levels of IL-33 and its receptor ST2 were investigated in the brain tissues of patients with AD and mouse models of AD. They found that IL-33/ST2 signaling was significantly downregulated in the brains of patients with AD and in mouse models and that this was associated with increased neuroinflammation and cognitive impairment. Furthermore, treatment with IL-33 or its agonist reduced neuroinflammation, improved synaptic plasticity, and alleviated cognitive impairment in the AD mouse model of AD. These findings suggest that IL-33/ST2 signaling plays a protective role in AD and could be a potential therapeutic target for treating the disease [73].

The effects of IL-33 treatment on AD pathology and cognitive function were investigated in a mouse model. IL-33 treatment could reduce the accumulation of Aβ plaques, neuroinflammation, and cognitive impairment in AD mouse models. Furthermore, the protective effects of IL-33 were mediated by the activation of microglia and astrocytes, which led to the clearance of Aβ plaques and reduction in neuroinflammation. These findings highlight the importance of IL-33 in the treatment of AD [74]. 

## 4. Neuromyelitis Optica

NMO is a CNS autoimmune inflammatory illness characterized by significant involvement of the optic nerves and spinal cord [75]. It is associated with a distinct pattern of astrocyte dysfunction and loss, which culminates in secondary demyelination and neurodegeneration. Before the discovery of the aquaporin-4 (AQP4) water channel, clarity was lacking on whether NMO was a distinct illness or a more severe type of multiple sclerosis [76]. NMO-IgG, which acts against AQP4, is the disease-specific autoantibody that plays a substantial role in the pathogenesis of NMO, as opposed to MS [10]. It specifically interacts with AQP4, which is present throughout the brain but accumulates in the spinal cord and optic neurons [77]. AQP4 is important for water homeostasis in the CNS. In the CNS, AQP4-IgG binds to AQP4 on astrocyte feet-ends, initiates complement-mediated astrocyte injury, and ultimately disrupts the blood-brain barrier and loss of astrocytes with demyelination [78]. Several inflammatory mediators, including interleukins (which are IL-6, IL-17, IL-21, and IL-36) are involved in the pathology of NMO.

The development and progression of NMO may be facilitated by ILs’ ability to both modulate the immune response and promote inflammation in the CNS. NMO is characterized by inflammation, demyelination, and tissue destruction in CNS. The abnormal regulation of ILs like IL-6 can contribute to the activation of the immune response and promotion of plasmablasts’ survival, which is a main source for the production of autoantibodies against AQP4. One of the mechanisms by which IL-17 contributes to the development of NMO is its role in the activation of T cells and the generation of pro-inflammatory cytokines that promote inflammation and demyelination in CNS [79].

**Interleukin-6:** The IL-6 pathway has increasingly been highlighted as a pathogenic mechanism of NMO. IL-6 interacts with membrane-bound or soluble IL-6 receptors before complexing with the transmembrane protein glycoprotein 130 to produce intracellular signals. IL-6, formerly known as B cell-stimulating factor 2, is a recognized plasma cell growth factor. It affects AQP4 antibody-secreting plasmablasts and increases antibody production from B cells, particularly plasmablasts, and helper T cell proliferation and differentiation [80]. AQP4-Abs are mostly IgG1 isotypes. AQP4-Abs promote IL-6 synthesis in AQP4-expressing astrocytes, and IL-6 signaling in endothelial cells decreases blood-brain barrier function [81]. When AQP4-Abs bind to the extracellular domain of the AQP4 receptor, they cause complement- and cell-mediated astrocytic injury and internalization of the glutamate transporter EAAT-2. Astrocytes are rendered ineffective, eventually leading to the removal of support for the surrounding cells, including oligodendrocytes and neurons. Granulocyte infiltration was accompanied by oligodendrocyte destruction and demyelination [82].

NMO and MS are believed to be immunologically distinct with different levels of cytokines and chemokines. A study was conducted to determine and compare the concentrations of different cytokines and chemokines in NMO (31), MS (29), and other non-inflammatory neurological disorders, including OND (18). CSF samples showed a higher concentration of IL-6 in patients (757.3 pg/mL) than in patients with MS and OND. A significant level of IL-6 was found in the serum sample of NMO patients compared to that in OND patients (*p* = 0.003), while the IL-6 CSF/Serum ratio was significantly higher in NMO patients (mean ± SD, 1255.1 ± 2596.4) compared to that in OND patients (mean ± SD, 12.3 ± 8.7) (*p* < 0.001), indicating that IL-6 has major role in pathogenesis of NMO [83]. This higher level of IL-6 in CSF samples of NMO patients compared to MS was also supported by another study, where the IL-6 level was found to be remarkably higher than that in MS and other neurological diseases (*p* < 0.001) [84]. This significantly higher level of IL-6 in NMO patients may be useful in the differential diagnosis of NMO and MS.

Iicoz et al. conducted a study to determine CSF and serum IL-6 levels in patients with NMO (23), optic neuritis (16), transverse myelitis (11), relapsing-remitting MS (27), and normal individuals (20). Higher IL-6 levels were observed in the serum and CSF samples of patients with NMO and transverse myelitis than in the other groups. Particularly, CSF IL-6 levels correlated with anti-AQP-4 levels and disease severity in NMO patients, and anti-AQP-4 positive NMO patients had higher serum/CSF IL-6 levels than anti-Aqp-4 negative patients [85]. This finding implies that IL-6 is involved in NMO pathogenesis, most likely through anti-AQP-4 related mechanisms. A two-year clinical trial was conducted on 81 NMO patients (seropositive or seronegative for AQP4-IgG), where approximately half were administered satralizumab (IL-6 receptor blocking antibody) and half were kept on placebo, in addition to stable immunosuppressive therapy. In patients with NMO, when combined with immunosuppressants, satralizumab reduced the risk of relapse compared to a placebo by blocking the IL-6 receptor, showing the involvement of IL-6 in NMO [86]. IL-6, which was found to be elevated in NMO, was shown to improve PB survival as well as AQP4-Ab secretion; however, blocking IL-6 receptor (IL-6R) signaling using an anti-IL-6R antibody decreased PB survival in vitro. These findings suggest that the IL-6-dependent B cell subset contributes to the development of NMO and offers a therapeutic approach to inhibit IL-6R signaling [80].

**Interleukin 17:** Th17 cells and IL-17-secreting CD8+ T cells release IL-17A, a potent pro-inflammatory cytokine. Promoting the chemokine-related recruitment of neutrophils and monocytes to the inflammatory area contributes to the pathogenesis of autoimmune diseases and presumably promotes delayed-type inflammation [38]. Th17-related cytokines were discovered in active lesions and reported to be elevated in the blood and CSF of patients with NMO, suggesting their role in the pathophysiology of the disease [87].

Th17 and IL-17 secreting CD8(+) T cells were studied in NMO (14), MS (20) patients, and control (16) individuals for one year. Th17 cells and IL-17-secreting CD8(+) T cells were counted by flow cytometry, and serum levels of IL-17 were evaluated via ELISA. Using flow cytometry, NMO patients were found to have more Th17 cells compared to patients with MS (3.72 vs. 2.58, *p* < 0.02) and control (*p* < 0.001). The IL-17-secreting CD8(+) T cell counts in NMO patients were greater than those in patients with MS (1.61 vs. 1.09%, *p* < 0.036) and control (*p* < 0.001). Serum IL-17 levels were higher in patients with NMO and MS than in control patients. IL-17-secreting CD8(+) T cells are elevated in patients with NMO and MS during relapse and play a role in the pathogenesis of both diseases [88].

Previously, 26 NMO patients were selected to observe the severity of NMO for the duration of a year via checking the level and presence of different cytokines, chemokines, and anti-AQP4 antibodies in NMO patients. Of the NMO patients, 62% showed antibodies against AQP4, while all patients had significantly higher levels of different cytokines, including IL-17, regardless of AQP4 antibody status, compared to the control group. This has been found to correlate with disease severity [89]. Kang et al. collected and checked serum samples from optic neuritis (ON) patients for the presence/absence of MOG-IgG-positive and IL-17 and their association with disease severity and compared them with a control group. Th17 cell-related cytokines/chemokines were significantly elevated in the sera of MOG-IgG-positive ON patients compared to those of MOG-IgG-negative ON patients. Serum levels of IL-17 and IL-21 were considerably elevated in MOG-IgG-positive ON patients compared with those in all other groups. According to the statistical analysis, the serum concentrations of MOG-IgG in ON patients were positively correlated with IL17 level. This demonstrates the role of IL-17 in the pathology of ON [90].

In another study, researchers found elevated levels of serum IL-17 in NMO patients compared to controls (*p* < 0.001) via ELISA, which was correlated with disease severity [91]. Therefore, inflammatory markers such as IL-17 can be used to diagnose NMO.

**Interleukin-36:** Recently, patients with NMO were found to show elevated blood levels of IL-36 agonists.

In this study, we demonstrated that neutrophil-derived IL-36 may induce microglia to generate neutrophil-stimulating cytokines, which in turn may promote neutrophil recruitment and hence contribute to neuroinflammation. The polymerization of defective neutrophils has been identified in NMO lesions, and suppression of neutrophilic proteases may reduce AQP4-IgG damage in the rat brain, suggesting that neutrophils play a significant role in the pathogenic mechanisms of NMO. CD4+T lymphocytes and BMDCs from mouse bone marrow express IL-36R and are responsive to IL-36. This suggests that elevated IL-36 levels affect immune cells in NMOSD, leading to persistent inflammation. The cytokine milieu in NMOSD patients with NMOSD was similar to that in the synovial fluid of patients with rheumatoid arthritis [11].

Different parameters (serum and CSF samples) were analyzed in a study that included 73 patients who had been diagnosed with NMO and ONND (other non-inflammatory neurological diseases), which served as controls. Neurological function was measured using the expanded disability status scale (EDSS), and the annual relapse rate (ARR) was calculated. Serum and CSF samples were analyzed for IL-36 levels using ELISA, and the results showed that IL-36 levels were considerably higher in patients with NMOSD than in controls. The EDSS score was also strongly linked with IL-36 levels in serum and CSF. The levels of IL-36 in the CSF were positively associated with those of leukocytes, proteins, and immunoglobulin IgG, suggesting that IL-36 could be a potential biomarker for tracking illness progression in NMO [92].

In another study, IL-36α, IL-36β, and IL-36γ levels were determined in the serum of NMO patients (50) and healthy control (HC) (30), and the correlations were assessed with different clinical characteristics. Patients with NMO had significantly higher serum levels of IL-36 and IL-36 compared to HCs (*p* = 0.005, *p* = 0.0001, respectively), notably during the acute period. Serum IL-36 levels in AQP4-IgG-negative NMO patients were substantially higher than those in HCs (*p* = 0.001), but significantly lower than those in AQP4-IgG-positive NMO patients (*p* = 0.040). Additionally, we discovered that the levels of serum IL-36 and IL-36 were significantly lower throughout the remission period compared to the acute phase (*p* = 0.0001 and 0.013, respectively). In patients with NMO, serum IL-36 levels were lower during the remission phase than during the acute phase. Serum IL-36 levels and ARR were shown to be significantly positively correlated (r = 0.698, *p* = 0.0001). We discovered a favorable correlation between blood IL-36 levels and the length of newly discovered spinal cord lesions and the EDSS scores at the lowest point in individuals with NMO (r = 0.613, *p* = 0.001; r = 0.426, *p* = 0.030). These findings imply that IL-36 may be crucial in the inflammatory etiology of NMO [93].

## 5. Autoimmune Encephalitis

Autoimmune encephalitis refers to a set of diseases characterized by inflammation and damage to brain tissue that manifest in a wide range of neurological symptoms. Autoantibodies are at fault, as they wrongly assault healthy brain cells, causing malfunction and damage. Recent research has led to advances in the identification of novel autoantibodies and the enhancement of diagnostic and treatment for autoimmune encephalitis [94]. Several subtypes of autoimmune encephalitis have been identified, each of which is associated with a distinct autoantibody. Anti-leucine-rich glioma-inactivated 1 (LGI1) encephalitis, anti-N-methyl-D-aspartate (NMDA) receptor encephalitis, and anti-contactin-associated protein-like 2 (CASPR2) encephalitis are only a few of the more prevalent varieties. Different types of autoimmune encephalitis present with a wide range of symptoms. Amnesia, seizures, personality shifts, autonomic instability, and mobility abnormalities are typical manifestations. Prompt diagnosis is crucial for effective therapy, especially given the possibility for irreversible brain damage if a diagnosis is delayed or missed [95].

**Interleukin-6:** IL-6 is thought to play a crucial role in the inflammatory response that occurs in the brain in autoimmune encephalitis [96]. In addition to immune cells like T cells and macrophages, non-immune cells like neurons and astrocytes also produce IL-6. Autoimmune encephalitis is caused by autoantibodies directed against brain proteins, which activate immune cells and cause the release of inflammatory cytokines like interleukin-6, Il-17, and Il-1β [97].

A study looked into the involvement of IL-6 and TNF-α in the etiology of autoimmune anti-NMDAR encephalitis, as well as their effect on learning and memory. The researchers discovered that IL-6 levels in the CSF of patients with anti-NMDAR encephalitis were substantially greater than in healthy controls (*p* < 0.01). Furthermore, IL-6 levels were found to be significantly associated with the severity of disease symptoms, such as memory impairments and hippocampus dysfunction (*p* < 0.01). The researchers also discovered that immunotherapy, such as steroids, intravenous immunoglobulin, and plasmapheresis, significantly lowered IL-6 levels in the CSF (*p* < 0.01). These results reveal that IL-6 may be involved in the cause of autoimmune anti-NMDAR encephalitis as well as its impact on learning and memory. Immunotherapy’s ability to reduce IL-6 levels provides a potential therapeutic target for the treatment of this medical condition [98].

Lee et al. examined tocilizumab’s efficacy to treat rituximab-resistant autoimmune encephalitis. A total of eighteen autoimmune encephalitis patients who had failed rituximab or relapsed were enrolled in the trial. These patients received tocilizumab. A total of 14 of 18 patients (77.8%) showed clinical improvement with tocilizumab. Seizures, mental symptoms, and cognitive impairment improved dramatically during the experiment. After receiving tocilizumab, CSF IL-6 levels dropped significantly. Tocilizumab individuals had no major side effects, according to the study. Tocilizumab treated auto-immune encephalitis in rituximab-resistant patients. According to the study, targeting IL-6 with tocilizumab may treat autoimmune encephalitis [99].

**Interleukin-17:** The cytokine IL-17 is pivotal in the development of autoimmune encephalitis. Th17 cells generate IL-17, which enhances the recruitment of inflammatory cells to the CNS in autoimmune encephalitis. IL-17 can also cause neuronal injury by activating inflammatory pathways and stimulating astrocytes and microglia to generate pro-inflammatory cytokines. 

A study showed that IL-17A levels may predict the severity of illness in non-NMDA-receptor autoimmune encephalitis (NRAE). IL-17A levels in CSF were tested in 50 NRAE patients within 48 h of hospital admission. Mild to moderate (*n* = 28) to severe (*n* = 22) disease severity groups were created. The study comprised 20 healthy controls. NRAE patients had greater CSF IL-17A levels than controls (*p* < 0.001). Severe NRAE patients had greater CSF IL-17A levels than mild to moderate patients (*p* < 0.001). IL-17A levels in the CSF were strongly linked with disease severity (*p* < 0.001) and could predict NRAE severity with 91% sensitivity and 86% specificity. These data show that IL-17A may contribute to NRAE development and may be a biomarker for disease severity. IL-17A’s clinical value as a predictive biomarker in NRAE must be confirmed in larger investigations [100].

A study indicated that poor-prognosis anti-NMDAR encephalitis patients had a higher CSF Th17 cell percentage than those with a positive prognosis. Poor prognosis patients had 7.91% CSF Th17 cells, while good prognosis patients had 2.31%. Its *p*-value was less than 0.001. Th17 cells in the CSF of anti-NMDAR encephalitis patients imply an involvement in disease development. Th17 cells release IL-17, which promotes inflammation and autoimmune. In anti-NMDAR encephalitis, Th17 cells may cause neuroinflammation and neuronal injury. In patients with a poor prognosis, Th17 cells and IL-17 may be therapeutic targets for anti-NMDAR encephalitis. To develop effective therapeutics targeting Th17 cells and IL-17, more research is needed [101].

**Interleukin-10:** IL-10 is a cytokine that controls immune responses by dampening inflammation. Therapeutic effects of IL-10 on lowering inflammation and enhancing neuroprotection have been investigated in the context of autoimmune encephalitis.

In an EAE mouse model, IL-10-transduced neural stem/progenitor cells (NSPCs) were tested for therapeutic potential. In mice, intravenous IL-10 transduced NSPCs prevented EAE. Compared to the control group, mice receiving IL-10 transduced NSPCs had fewer clinical symptoms. Clinical symptoms were scored on a 0–5 scale with a maximum value of 5. IL-10 transduced NSPCs had an average clinical score of 1.55, while the control group had 2.85. In mice with EAE, IL-10 transduced NSPCs reduced CNS inflammation. This was measured by CNS pro-inflammatory cytokines. The control group had 127.5 pg/mL of IFN-γ in the CNS, while the IL-10 transduced NSPCs group had 70.1. TNF-α levels dropped from 77.5 pg/mL in the control group to 39.5 in the IL-10 transduced NSPCs group. In mice with EAE, IL-10-transduced NSPCs reduced CNS demyelination. Demyelination was measured by CNS myelin loss. Myelin loss averaged 34.5% in the control group and 19.7% in the IL-10 transduced NSPCs group. According to the study, IL-10 transduced NSPCs reduce pro-inflammatory cytokines and induce CNS regulatory T cells. These data imply that stem cell-based therapy, specifically NSPCs, may be a potential treatment for autoimmune encephalomyelitis and other neuroinflammatory illnesses [102].

A new probiotic blend was tested for treating EAE in mice by Lavasani et al. It was shown that IL-10-producing regulatory T cells in the probiotic mixture suppressed inflammation and prevented EAE progression. The probiotic mixture-treated mice had fewer clinical signs than the control group. Clinical symptoms were scored on a 0–5 scale with a maximum value of 5. The probiotic combination group had an average clinical score of 0.9, while the control group had 2.2. EAE animals’ CNS inflammation was decreased by the probiotic mixture. CNS pro-inflammatory cytokines served as a gauge for this. The probiotic mixture group had 96.3 pg/mL IFN-γ in the CNS, compared to 192.2 in the control group. TNF-α levels dropped from 133.9 pg/mL in the control group to 58.4 in the probiotic mixture group. The probiotic cocktail increased IL-10-producing regulatory T cells in mice with EAE’s CNS. Compared to the control group, the probiotic mixture group had more CNS regulatory T cells that produced IL-10. In mice with EAE, the probiotic cocktail changed the intestinal microbiota by increasing the proportion of beneficial bacteria like Bifidobacterium and Lactobacillus while decreasing the proportion of harmful bacteria like Escherichia coli. According to this study, the effectiveness of the probiotic blend in autoimmune encephalomyelitis is due to IL-10-producing regulatory T cells that decrease inflammation and prevent EAE progression [103].

**Figure 2:**
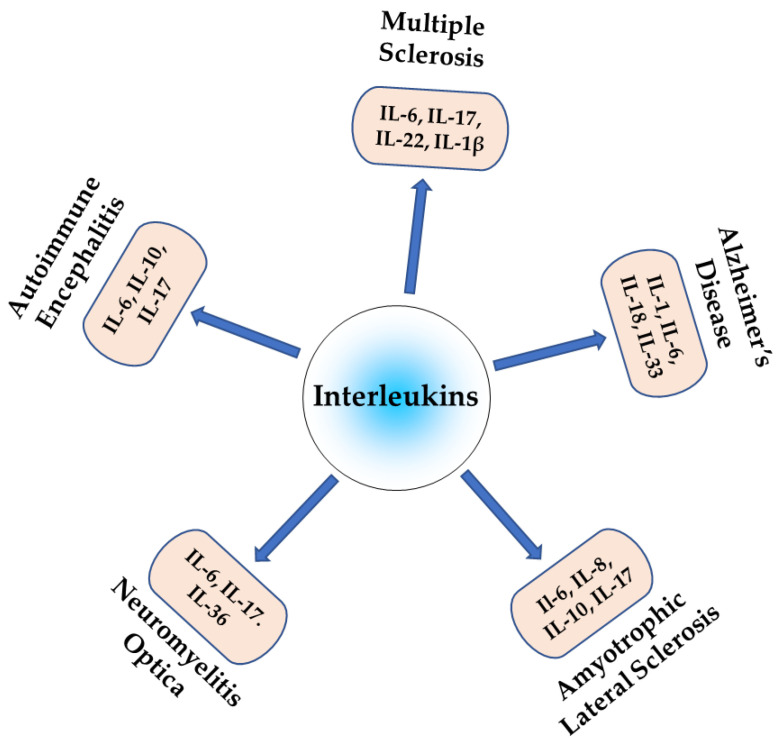
Role of different interleukins in autoimmune neuroinflammatory diseases.

Neuroinflammation is a complex process that is crucial in the pathogenesis of autoimmune neurological diseases. ILs are key regulators of neuroinflammation and, therefore, also influence pathogenesis. Pro-inflammatory ILs can activate microglia and astrocytes, leading to the release of additional pro-inflammatory cytokines and chemokines, which can exacerbate neuroinflammation and contribute to neurodegeneration, while anti-inflammatory interleukins suppress neuroinflammation and promote neuronal survival. The dysregulation of the balance between pro- and anti-inflammatory interleukins can lead to chronic neuroinflammatory diseases, as shown in Figure 2, which promotes neurodegeneration and cognitive decline. Understanding the role of specific ILs in neuroinflammation may reveal novel therapeutic targets for treating these debilitating disorders. Further research is required to fully elucidate the specific mechanisms via which interleukins contribute to neuroinflammation and to develop targeted therapies to modulate interleukin signaling in the CNS. 

## Figures and Tables

**Table 1:** Interleukins and their mechanisms in neuroinflammation.

Interleukin	Types	Mechanism in Neuroinflammation	Reference
**Pro-inflammatory**	IL-1β	Induces neuroinflammation, promotes neuronal damage and death through activation of the inflammasome and downstream signaling pathways	[7]
	IL-6	Promotes neuroinflammation and mediates blood-brain barrier disruption through activation of the JAK-STAT signaling pathway	[8]
	IL-17	Induces neuroinflammation and promotes tissue damage by activating pro-inflammatory signaling pathways and promoting the recruitment of neutrophils	[9]
	IL-23	Induces neuroinflammation and promotes tissue damage by promoting the differentiation of Th17 cells and production of pro-inflammatory cytokines	[10]
	IL-36	Induces neuroinflammation and promotes the production of pro-inflammatory cytokines, chemokines, and adhesion molecules	[11]
**Anti-inflammatory**	IL-2	Promotes neuroprotection and repair by enhancing the survival and differentiation of neural stem cells and neurons	[12]
	IL-4	Reduces neuroinflammation and promotes neuroprotection by inhibiting the production of pro-inflammatory cytokines and enhancing anti-inflammatory cytokine production	[13]
	IL-10	Inhibits neuroinflammation and promotes neuroprotection and repair by inhibiting the production of pro-inflammatory cytokines and promoting the differentiation of regulatory T cells	[14]
	IL-27	Inhibits neuroinflammation and promotes neuroprotection and repair by inhibiting the production of pro-inflammatory cytokines and promoting the differentiation of type 1 regulatory T cells	[15]
	IL-35	Inhibits neuroinflammation and promotes neuroprotection and repair by inhibiting the production of pro-inflammatory cytokines and promoting the differentiation of regulatory T cells	[16]

**Table 2:** Assessment of interleukin targets and their clinical correlations when treating pain and inflammation associated with nervous system diseases.

Clinical Drugs	Target Interleukin	Site of Action	Disease Model
Canakinumab	IL-1β	Periphery, brain, spine	Knock-out mice, peripheral nerve injury
Tocilizumab	IL-6	Periphery, spine	CCI, peripheral nerve injury, knock-out mice
Secukinumab	IL-17	Periphery	Peripheral nerve injury, chemical injection, knock-out mice, arthritis
Glatiramer	IL-4	Periphery	CCI, partial nerve injury
Calcineurin	IL-10	Periphery, brain	Partial/complete nerve injury, neuritis

## Data Availability

Not applicable.
